# A novel GH3-β-glucosidase from soda lake metagenomic libraries with desirable properties for biomass degradation

**DOI:** 10.1038/s41598-024-60645-y

**Published:** 2024-05-01

**Authors:** Oliyad Jeilu, Erik Alexandersson, Eva Johansson, Addis Simachew, Amare Gessesse

**Affiliations:** 1 Department of Plant Breeding, Swedish University of Agricultural Sciences, Box 190, 23422 Lomma, Sweden; 2 Department of Civil and Environmental Engineering, Northwestern University, Evanston, IL 60208 USA; 3 Institute of Biotechnology, Addis Ababa University, P O Box 1176, Addis Ababa, Ethiopia; 4 Department of Biological Sciences and Biotechnology, Botswana International University of Science and Technology, Private Bag 16, Palapye, Botswana

**Keywords:** Beta-glucosidase, Glycoside hydrolase family 3 (GH3), Soda lakes, Enzyme characterisation, Environmental biotechnology, Industrial microbiology, Enzymes, Microbiology

## Abstract

Beta-glucosidases catalyze the hydrolysis of the glycosidic bonds of cellobiose, producing glucose, which is a rate-limiting step in cellulose biomass degradation. In industrial processes, β-glucosidases that are tolerant to glucose and stable under harsh industrial reaction conditions are required for efficient cellulose hydrolysis. In this study, we report the molecular cloning, *Escherichia coli* expression, and functional characterization of a β-glucosidase from the gene, CelGH3_f17, identified from metagenomics libraries of an Ethiopian soda lake. The CelGH3_f17 gene sequence contains a glycoside hydrolase family 3 catalytic domain (GH3). The heterologous expressed and purified enzyme exhibited optimal activity at 50 °C and pH 8.5. In addition, supplementation of 1 M salt and 300 mM glucose enhanced the β-glucosidase activity. Most of the metal ions and organic solvents tested did not affect the β-glucosidase activity. However, Cu^2+^ and Mn^2+^ ions, Mercaptoethanol and Triton X-100 reduce the activity of the enzyme. The studied β-glucosidase enzyme has multiple industrially desirable properties including thermostability, and alkaline, salt, and glucose tolerance.

## Introduction

Glycoside Hydrolase Family 3 (GH3), particularly beta-glucosidases, are pivotal in a variety of biological processes due to their ability to hydrolyze glycosidic bonds in complex sugars. These enzymes are known for their substrate versatility, playing a significant role in carbohydrate metabolism^1^. GH3 contains enzymes that can hydrolyze the terminal glycosidic bond in the non-reducing end of various glycosides and glycoconjugates^2,3^. GH3 enzymes are of interest for industrial applications^3^, and their structure, specificity, and biological role have been found to be extraordinarily diverse. Members of this family have been demonstrated as having a variety of enzymatic activities, including those of β-glucosidase, β-xylosidase, α-L-arabinofuranosidase, and N-acetyl β-glucosaminidase^3,4^. GH3 enzymes have been widely found in bacteria, fungi, and plants^5^. Thus, GH3 is one of the most prevalent families of carbohydrate-active enzymes.

The β-glucosidases of the GH3 are known to hydrolyz terminal non-reducing β-D-glucosidic linkages, primarily cleaving cellobiose into glucose^6,7^. These enzymatic activities are of potential use in numerous biotechnological processes, including biomass degradation, pharmaceutical production, and the food industry^8,9^. For the degradation of cellulosic biomass, the β-glucosidase activity is regarded as the rate-limiting factor^9,10^.

To increase substrate accessibility in many industrial processes, including biomass degaradation, the β-glucosidases need to operate under extreme conditions. Thus, enzymes like β-glucosidases must withstand high temperatures, extreme pH levels, elevated substrate and product concentrations, and the presence of organic solvents or detergents. These factors, prevalent in industries such as biofuel production, can significantly impact enzyme structure and function, necessitating robust enzymes capable of maintaining their activity^11–13^. Unfortunately, glucose which is the desired end product of biomass degradation, is known to inhibit and limit the activity of the β-glucosidases^14^. As a result, β-glucosidases that are stable both under harsh industrial conditions^15,16^ and high glucose concentrations are required^10^. Consequently, most of the current industrially important enzymes, including the β-glucosidases, originate from mesophilic microorganisms. Despite their numerous benefits, these enzymes frequently lack stability under harsh industrial processes^11^. As a result, there is a high need for the exploration of more stable enzymes from extremophiles.

One source of new enzymes for industrial applications are extremophiles, which are able to live in harsh environments such as soda lakes^17,18^. Soda lakes present a variety of physical and chemical extremes, including alkalinity, and salinity (NaCl)^19^. Some of the are found in the East African Rift Valley in Ethiopia and Kenya^20,21^. Despite the particular environment, soda lakes are known to be incredibly productive and to harbour a range of haloalkaliphilic microbial communities that are responsible for the cycling of essential elements such as carbon, nitrogen, and sulfur^22–24^. These microorganisms offer huge potential as sources of biocatalysts that work under extreme conditions, useful for conditions in many industrial operations^25,26^.

To discover novel biocatalytic enzymes with potentially high functionality in industry, appropriate approaches are needed for screening and evaluation of a large number of microorganisms from extreme environments. Harnessing the microbial genetic pool in any environment, is possible by isolating a given microbial strain in pure culture form, followed by screening for enzyme production and cloning of the gene for further manipulation and/or large-scale production^27–29^. Even though this culture-dependent approach provides many novel microorganisms, only a small fraction of microbes (according to many estimates ≤ 1%) can be recovered in culture media^30,31^.

Recently, culture-independent methods such as metagenomics have provided a powerful tool to access the genetic and metabolic diversity of microorganisms in any environment, which overcomes the limitations of culture-dependent approaches^32^. In functional metagenomics, genes are isolated from microorganisms of a certain environment and then cloned into libraries. Thus, functional metagenomics is favourable over other commonly used methods, as it allows the detection of microorganisms that are not culturable^33,34^. Functional metagenomics has already been extremely useful and effectively employed to identify enzymes with specific functions and novel enzyme families, including glycoside hydrolases family 3^4,35,36^. From the constructed library, a single gene or a group of genes are then identified based on certain enzymatic activities. The gene products are thereafter further assessed using a suitable screening assay^11^.

Based on our previous study, the aim of this study was to investigate and characterize the enzymatic properties of a gene (CelGH3_f17) previously identified in a metagenomic library from an Ethiopian soda lake^37^, to elucidate its potential application in industrial processes. This included the cloning and expression of the gene, to study the properties and stability of the encoded β-glucosidase enzyme under extreme industrial conditions, such as high temperatures and varying pH levels. The study aimed to assess the enzyme's efficiency in biomass degradation and its resistance to inhibition by glucose, thereby contributing to the development of more robust biocatalysts for biomass degradation.

## Results

### Identification and selection of CelGH3_f17

In our preceding research^37^, we constructed metagenomic libraries from Ethiopian Soda lakes, which underwent a functional screening process targeting hydrolytic enzymes, including β-glucosidase activity. Subsequent to the screening, clones demonstrating positive results were chosen for sequencing. These sequenced clones then underwent comprehensive bioinformatics analysis and were annotated utilizing the carbohydrate-active enzyme (CAZy) database^37^. Among the genes identified through this process, a gene named CelGH3_f17, was chosen for more detailed downstream analysis due to its higher identity percentage and cloning success.

### Sequence and structural analysis of the CelGH3_f17 protein

**Figure 1:**
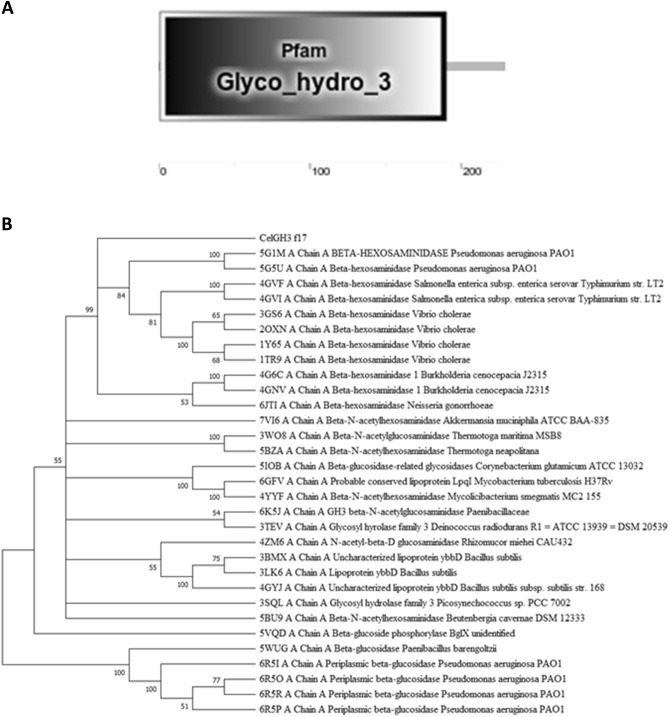
Sequence analysis of CelGH3_f17 (**A**) The predicted modular architecture of CelGH3_f17. (**B**) The phylogenetic tree was constructed using the Neighbor-Joining method^80^, based on the blast analysis of CelGH3_f17 with 31 amino acid sequences of proteins in the protein data bank. Branch reliability is indicated by the percentage of 500 bootstrap replicates supporting each cluster of taxa next to the branches. The evolutionary study was performed using MEGA11 software^75^.

CelGH3_f17 had an amino acid sequence of 229 and using the ExPASy’s Compute pI/MW tool (www.expasy.org/resources/compute-pi-mw) the theoretical isoelectric point and molecular mass of the protein encoded by CelGH3_f17 were 6.16 and ~ 25 kDa, respectively. The sequence analysis with the SMART software package indicated that the putative protein had one GH3 region in the C-terminal in the amino acid positions 1–190 (Fig. 1A). Conserved domain searches in NCBI confirmed that the CelGH3_f17 gene is likely to encode GH3 β-glucosidase/β-hexosaminidase. BLASTP showed that the CelGH3_f17 protein has a sequence similarity of 24–54% with 31 other proteins in the protein database (Supplementary Table S1). The majority of these proteins were classified as GH3 enzymes and were taxonomically linked to 21 organisms. Among them, 19 were identified as bacteria; where most of them were from *Pseudomonas spp*., one was traced back to a fungal family, and the origin of one remained unidentified (Fig. 1B). Nevertheless, the similarity ranging from 24 to 54% is relatively low, signifying the specific sequence of the CelGH3_f17 protein.

**Figure 2:**
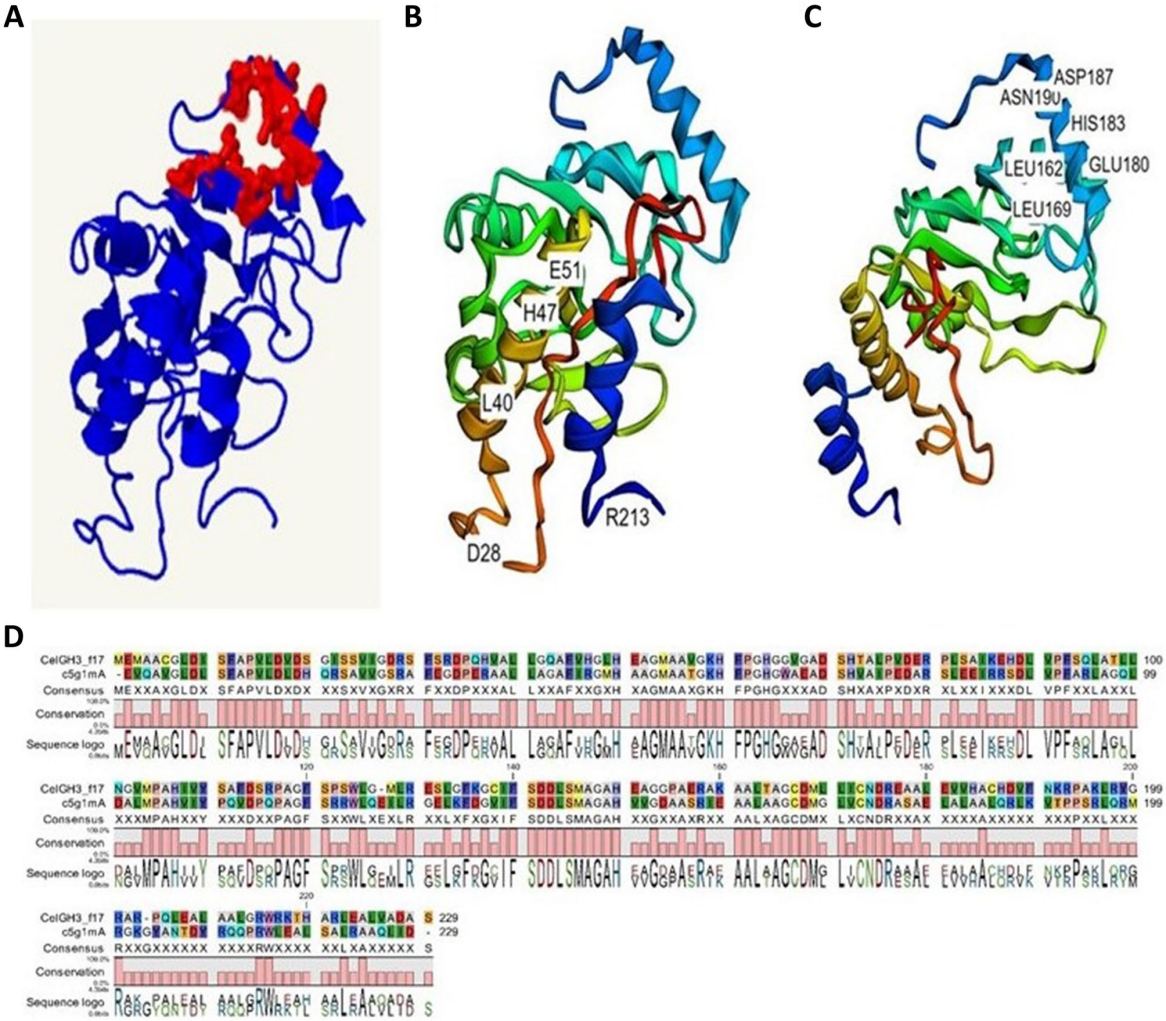
The predicted three-dimensional (3D) structural model of the CelGH3_f17 protein (**A**). The predicted active sites of the CelGH3_f17 protein (**B**). The predicted substrate recognition-pocket site of the CelGH3_f17 protein (**C**). The pairwise alignment of the CelGH3_f17 with c5g1mA, Crystal structure of NagZ from *P*. *aeruginosa* (**D**).

Phyre2 identified a total of 51 homologous templates with a confidence level of 100% (Supplementary Table S2). These templates exhibited a sequence identity ranging from 51% down to 15%. Among these templates, the crystal structure of NagZ from *Pseudomonas aeruginosa*^38^ was identified as the most closely related, sharing a sequence identity of 51% with the target protein. Consequently, this high-confidence template, NagZ, was utilized as the primary basis for modelling the three-dimensional structure of the CelGH3_f17 protein. The 214 residues (93%) of the CelGH3_f17 protein were aligned to the template crystal structure of NagZ (Fig. 2D). In the predicted structure of the CelGH3_f17 protein, the conserved amino acid residues of aspartic acid D187, glutamic acid E180, leucine L162, leucine L169, histidine H183, and asparagine N190 (Fig. 2A,C) form the pocket-shaped substrate recognition domain (Fig. 2C). The amino acid residues, aspartic acid D28, glutamic acid E51, arginine R213, and histidine H47 are predicted as the enzymatic active sites among the conserved amino acids (Fig. 2B). In addition, the aspartic acid D28 and glutamic acid E51 are the predicted catlytic active sites. The aspartic acid residue often acts as the nucleophile, and it is likely that E51 serves as the proton donor.

### Expression and purification of the CelGH3_f17 protein

**Figure 3:**
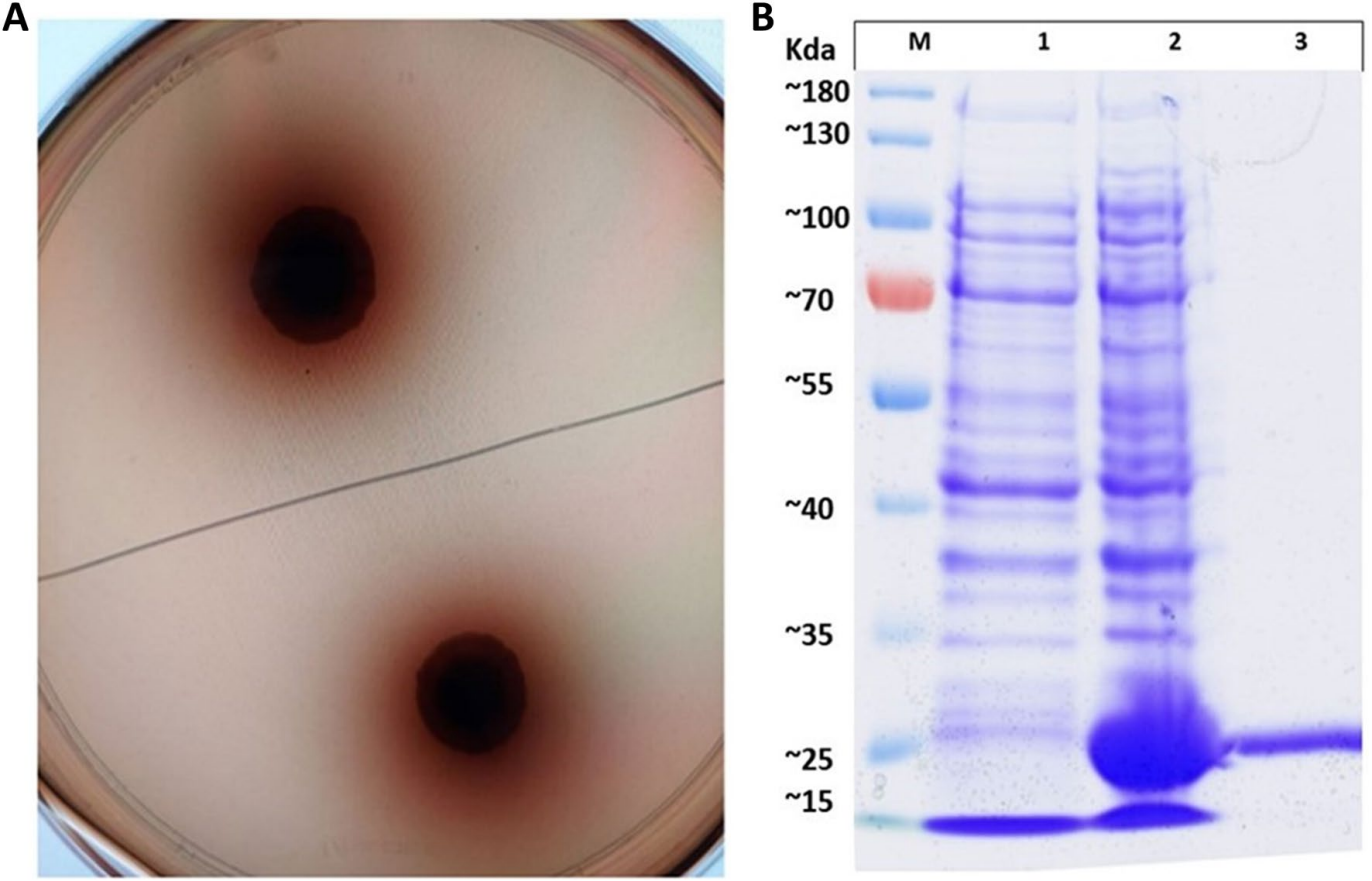
(**A**) Agar plate β-glucosidase assay of CelGH3_f17. (**B**) SDS-PAGE analysis of the purified, recombinant CelGH3_f17 protein. M, marker proteins; lane 1, IPTG-uninduced crude extract of BL21 (DE3) carrying pET-CelGH3_f17; lane 2, IPTG-induced crude extract of BL21 (DE3) carrying pET-CelGH3_f17; lane 3, Ni–NTA chromatography purified extract of BL21 (DE3) carrying pET-CelGH3_f17.

To characterize the biochemical properties of the CelGH3_f17 protein, it was heterologously expressed with an N-terminal His-tag fusion protein using the pET-28a expression system under the control of the T7 lac promoter in *E. coli* BL21. A black halo around the colonies of CelGH3_f17 on the agar plate indicated the presence of β-glucosidase activities (Fig. 3A).

The recombinant CelGH3_f17 protein was secreted and expressed in a soluble form. The molecular mass of the purified enzyme was estimated at approximately 25 kDa as a single band by SDS-PAGE, which is consistent with the predicted molecular weight (Fig. 3B).

### Characterization of the β-glucosidase activity of the CelGH3_f17 protein

**Figure 4:**
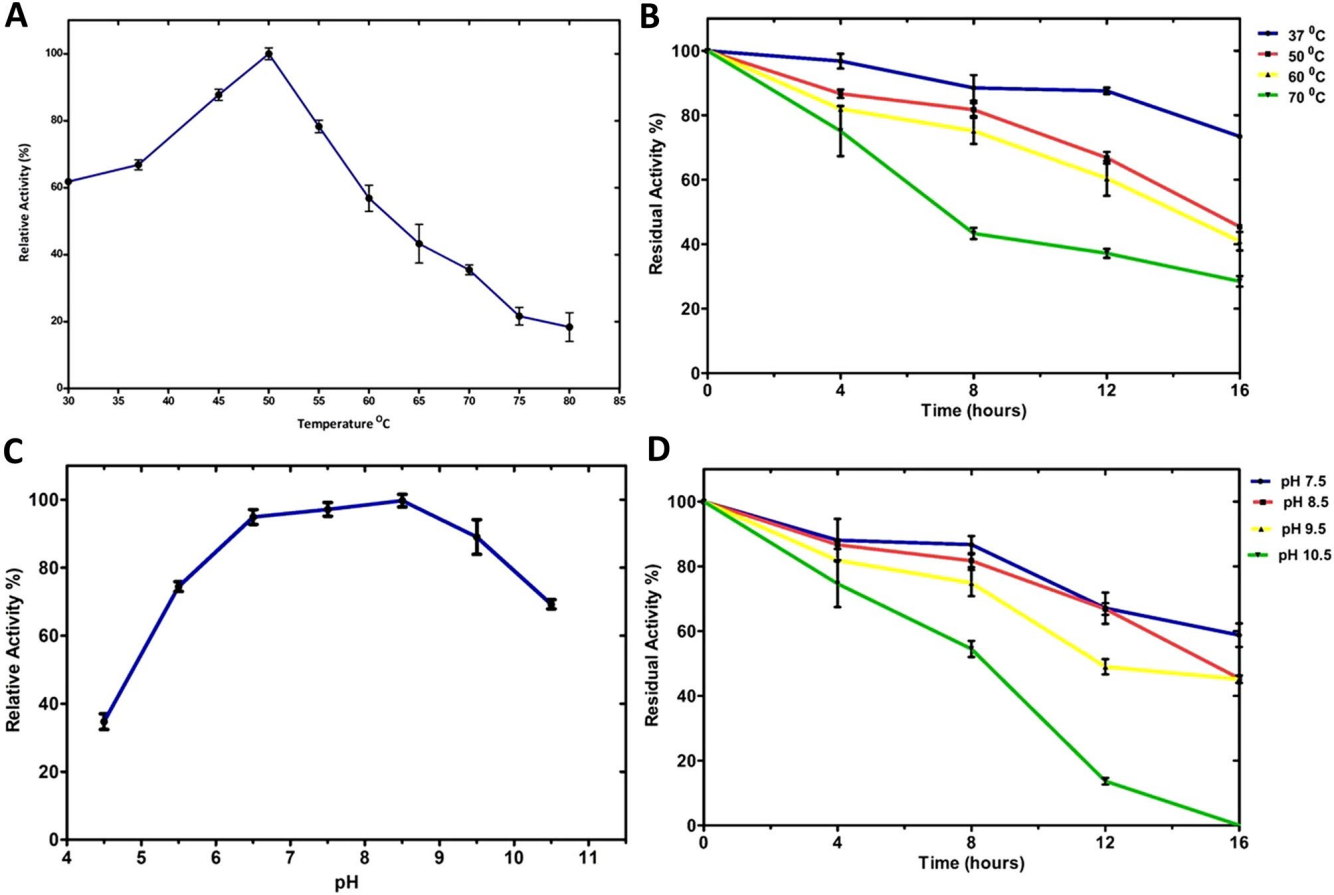
The CelGH3_f17’s β-glucosidase activity and stability analysis. (**A**) The relative activity of the enzyme across a range of temperatures, demonstrating optimal activity. (**B**) Residual activity of the enzyme over a 16-h period at various temperatures, demonstrating the stability (**C**) The relative activity of the enzyme across a range of pHs, demonstrating optimal activity. (**D**) Residual activity of the enzyme over a 16-h period at various pHs demonstrating the stability of CelGH3_f17 over time.

The enzyme encoded by CelGH3_f17 displayed its highest level of β-glucosidase activity at a temperature of 50 °C. Remarkably, even within a range spanning from 30 to 60 °C, this enzyme retained more than 50% of its relative activity (Fig. 4A). The enzyme's stability was assessed over time, and it exhibited high stability at 37 °C and 50 °C, as evidenced by the gradual decrease in residual activity over a 16 h period. At 70 °C, the enzyme's residual activity decreased significantly (Fig. 4B).

The purified β-glucosidase peak hydrolytic activity was observed at pH8.5. Furthermore, it was observed that this enzyme exhibited a substantial level of relative activity exceeding 70% across a broad pH range, reaching as high as pH 10.5 (Fig. 4C). Further investigation into the stability of β-glucosidase at various pH levels showed that the enzyme was most stable at pH 7.5 and 8.5, maintaining high activity over an 16-h period. In contrast, at 10.5, the enzyme's activity reduced significantly over time (Fig. 4D).

**Figure 5:**
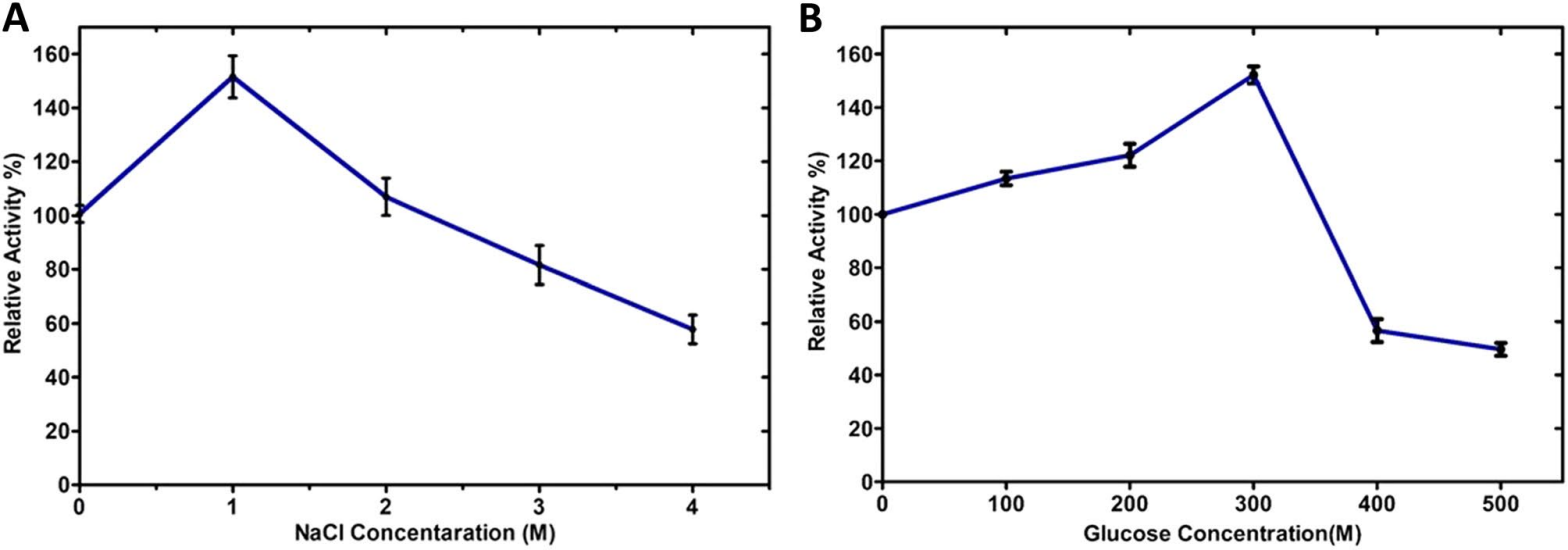
Effect of NaCl and glucose concentrations on the CelGH3_f17’s β–Glucosidase activity. (**A**) Relative enzyme activity at varying molar concentrations of NaCl. (**B**) Relative enzyme activity at different molar concentrations of glucose.

The β-glucosidase activity of the CelGH3_f17 was increased when supplemented with a salt concentration of 1 M (Fig. 5A). The enzyme still exhibited substantial activity, around 60% residual activity, even when exposed to a much higher salt concentration of 4 M. Furthermore, the β-glucosidase activity of the CelGH3_f17 showed a noticeable improvement when supplemented with glucose, with enhancements observed up to a concentration of 300 mM. However, beyond the threshold of 300 mM glucose, there was a sharp decline in the β-glucosidase activity of the enzyme (Fig. 5B).

The addition of Fe did not affect the activity of this β-glucosidase. However, Cu^2+^, Mg^2+^ and Mn^2+^ ions resulted in a decreased activity of β-glucosidase, while, Al^3+^ and K^1+^ ions had little impact on the activity of the enzyme (Table 1). Furthermore, Glycerol and SDS enhanced the β-glucosidase activity of the CelGH3_f17 protein, while, Mercaptoethanol and Triton X-100 decreased the activity considerably (Table 2).

**Table 1:** The effect of metal ions on the β-glucosidase activity of the CelGH3_f17.

Metal ions	Enzyme activity (U)	Relative activity (%)
Control	53.6 ± 0.4	100.0
CuCl_2_	25.7 ± 2.8*	48.0
FeCl_3_	53.2 ± 1.8	99.2
KCl	43.4 ± 6.2	80.9
CaCl_2_	37.6 ± 0.3*	70.0
ZnCl_2_	41.7 ± 3.0	77.7
MnCl_2_	23.7 ± 0.7*	44.2
MgSO_4_	35.3 ± 0.9*	65.8
AlCl_3_	48.3 ± 3.6	89.9

The enzyme activity is given with mean and standard error. Statistical significance between treatments and a control group was assessed using post-hoc Tukey's Honestly Significant Difference (HSD) test. Differences were considered statistically significant at a p-value of < 0.05.

*Significant difference of the treatement with control.

**Table 2:** The effect of surfactant and chelating agents on the β- activity of CelGH3_f17.

Surfactant and chelating agents(10mM)	Enzyme activity (U)	Relative activity (%)
Control	53.6 ± 0.4	100
EDTA	51.4 ± 1.3	96
SDS	76.6 ± 2.0*	143
DMSO	33.2 ± 2.2*	62
Glycerol	56.3 ± 0.6*	105
Mercaptoethanol	7.5 ± 0.1*	14
Triton X-100	12.8 ± 0.8*	24

The enzyme activity is given with mean and standard error. Statistical significance between treatments and a control group was assessed using post-hoc Tukey's Honestly Significant Difference (HSD) test. Differences were considered statistically significant at a p-value of < 0.05.

*Significant difference of the treatement with control.

CelGH3_f17 had the best hydrolyzing capacity against *p*NPG. CMC was hydrolyzed at 30%, but there was no hydrolytic activity on xylan (Table 3).

**Table 3:** Effects of the β-glucosidase on different substrates.

Substrate	Linkage of a glycosyl group	Enzyme activity (U)
*p*NPG	βGlc	53.6 ± 0.4
CMC	β (1,4) Glc	16.0 ± 1.5*
Xylan	β (1,3) Glc	0.0 ± 0.0*

The enzyme activity is given with mean and standard error. Statistical significance between treatments and a control group was assessed using post-hoc Tukey's Honestly Significant Difference (HSD) test. Differences were considered statistically significant at a p-value of < 0.05.

*Significant difference of the treatement with control.

## Discussion

Enzyme discovery via functional metagenomics, followed by biochemical characterization, is critical for linking genes and gene sequences to practical applications in industry^39^. Thus, in this study, we report the molecular cloning, expression in *E. coli*, and functional characterization of the gene product of the CelGH3_f17, which was isolated from our previous metagenomics libraries constructed from the Ethiopian soda lakes^37^.

The amino acid sequence analysis places CelGH3_f17 among the GH3, which includes β-glucosidases, N-acetylglucosaminidases, β-xylosidases, and α-arabinofuranosidases^5^. The CelGH3_f17 encoded for 229 amino acids corresponding to an approximate protein molecular weight of 25 kDa. The modest amino acid sequence length and lower molecular weight of CelGH3_f17 compared to, e.g., the Cen502 β-glucosidase identified from *Bursaphelenchus xylophilus*^9^, and a novel GH3 β-glucosidase^40^ illustrates the wide-ranging structural and functional diversity of GH3^41^. This particular attribute of CelGH3_f17 could be due to its evolutionary adaptation to the Ethiopian soda lake environment where it was discovered. To survive in extreme environments such as Soda lakes, microorganisms have evolved various adaptive strategies^42^, particularly in their protein structures and compositions. Enzymes such as CelGH3_f17, with their smaller size, may have developed to function optimally in such niches, possibly conferring benefits such as increased efficiency in their synthesis and maintenance^43–45^.

The sequence relatedness of CelGH3_f17 to the other proteins was low, with a maximum of 54% to beta-hexosaminidase from *Pseudomonas aeruginosa* PAO1^38^, where the lowest hit was 24% to periplasmic beta-glucosidase from *P. aeruginosa* PAO1^46^. This indicates that CelGH3_f17 might be a new enzyme. This distinctness could be due to a unique evolutionary path or to specific adaptations in its enzymatic functions. The active site of GH3 families is composed of a well-conserved pair of aspartic acid residues that act as catalytic nucleophile and glutamic acid residues that act as catalytic acid/base^4,5,47^. GH3 enzymes also have three additional highly conserved amino acid residues (Arg, Lys, and His) that are predicted to be involved in catalysis in addition to its two critical residues^4^. Despite the fact that CelGH3_f17 showed a low sequence relation to the other GH3 β-glucosidases, it shared some common features with other GH3 enzymes, e.g. a conserved aspartate residue which acts as a catalytic nucleophile across different species, and the position of the general acid/base residue, which is phylogenetically variable^41^. Furthermore, the crystal structure of NagZ in *P. aeruginosa*, which served as the model for the CelGH3_f17 enzyme, is a monomer. Based on this, we infer that CelGH3_f17 may also be monomeric. However, additional research is required to definitively determine its oligomeric state.

We used an agar plate that contained an esculin and ferric ammonium citrate to confirm the β-glucosidase activity of the cloned CelGH3_f17 gene. Esculin is comprised of a glucose molecule attached to esculetin that, when released by hydrolysis, combines with ferric ammonium citrate in the medium to generate a black precipitate. The black precipitate detected suggests the presence of glucosidase enzymatic activity^48^.

The fact that the recombinant CelGH3_f17 protein was secreted in soluble form is attractive for industrial applications, since purification becomes less expensive and time-consuming^49^. In addition, extracellular enzyme production is more effective than intracellular enzyme production since it improves protein folding and reduces proteolytic degradation^50,51^.

The CelGH3_f17 exhibited optimum activity in alkaline pHs with a maximum hydrolytic activity at pH 8.5 and temperature 50 °C. In line with other studies, the β-glucosidase, Cen502, was shown to exhibit optimal activity at 38 °C and pH 8.0, and is stable across a pH range of 7.0 to 9.0^9^. An enginerred β-glucosidase from a previous study was found with optimal activity at 75 °C and pH 4.5^52^.

The optimal temperature for CelGH3_f17 β-glucosidase is consistent with many β-glucosidases in literature and the wide range of temperature over which the enzyme retains significant activity suggests it may be robust for various biotechnological applications. The activity of enzymes in elevated temperatures is a crucial factor in the breakdown process of biomass^47^. However, the loss of stability at higher temperatures is a common characteristic among enzymes due to thermal denaturation, which affects the enzyme's tertiary and quaternary structures^53^. The CelGH3_f17 β-glucosidase had a higher activity and stability at alkaline pH compared to most other β-glucosidases characterized. Most β-glucosidases that have been identified to date exhibit maximal activity in the acidic pH range (pH 4–6.5), retaining only minimal activity or displaying instability in mildly alkaline conditions^54–57^. The CelGH3_f17 β-glucosidase's ability to retain considerable activity up to pH 10.5 indicates its potential usefulness in slightly alkaline conditions where other enzymes might not perform well. The reduced stability at higher pH values has in previous studies been attributed to the disruption of ionic bonds and electrostatic interactions within the enzyme's structure^52^.

β-glucosidases have great application prospects in many industrial fields, including aroma improvement of juice and wine, and lignocellulosic biomass conversion in biofuel production^58^. Typically, lignocellulosic biomass conversion takes place at high temperatures in the presence of alkaline conditions^59^. The neutralisation of these alkalines results in the formation of salts, which requires massive amounts of water and energy to be removed for subsequent processes to proceed. From previous studies, it is known that the alkaline conditions needed have a negative effect on the activity and the stability of most known enzymes^60^. For the enzyme reported here, the β-glucosidase activity remained consistent even when exposed to high salt concentrations, and the activity even increased with the addition of 1 M NaCl. This reflects that the CelGH3_f17 gene comes from metagenomic libraries derived from Ethiopian soda lakes, which hold extreme conditions characterized by high alkalinity and salinity^22,61^.

One commonly observed unfavourable characteristic that impedes the efficient breakdown of cellulose via enzymatic hydrolysis is glucose, which competitively inhibits the activity of β-glucosidase^58^. This results in the accumulation of cellobiose and oligosaccharides, which repress endoglucanase and exoglucanase activities, ultimately impeding the entire process of cellulose hydrolysis^14,50,62^. The majority of well-known GH3 are inhibited when exposed to glucose concentrations lower than 100 mM^58,63,64^. In this study, up to 300 mM of glucose rather increased the β-glucosidase activity of CelGH3_f17. This strong glucose tolerance offers an additional advantage in mitigating the catabolic inhibition imposed on endoglucanase and cellobiohydrolase due to the accumulation of cellobiose during the bioconversion process.

It has been proposed that metal ions are responsible for inhibiting β-glucosidases' catalytic activity^65^. Most of the supplemented metal ions did not have a significant impact on the activity of β-glucosidases evaluated here, with the exception of the Cu^2+^ and Mn^2+^ ions. However, these metal ions have been found to significantly inhibit the activity of β-glucosidases evaluated in previous studies as well^14,66,67^. This result implies that the CelGH3_f17 protein active catalytic sites may include thiol groups, which may be necessary for the preservation of the 3D structure of the active protein. Disulfide bonds are crucial to maintain the natural shape of a protein thereby controlling its biological activity^68–70^. Disulfide bonds can be chemically reduced by reducing agents like mercaptoethanol, and these modifications may result in changes in the structure of the protein, which have an impact on their activity^68^. Indeed, mercaptoethanol decreased the β-glucosidases which indicates that disulfide bonds are essential for the activity of the β-glucosidases of CelGH3_f17. Moreover, the fact that the chelating chemical EDTA did not affect the activity of β-glucosidase indicates that the CelGH3_f17 protein is not a metalloprotein.

The moderate sequence similarity of the CelGH3_f17 protein to other known GH3 enzymes indicates that the CelGH3_f17 protein may possess unique structural features conducive to its stability and activity profile. The structural modeling based on the homologous NagZ^71^ suggests that CelGH3_f17 has a well-defined active site with specific amino acids contributing to its catalytic process. This information could be valuable for further enzyme engineering efforts aimed at enhancing the desirable traits of CelGH3_f17. The β-glucosidase enzyme of CelGH3_f17, with optimal activity at 50 °C, pH 8.5, and enhanced activity in the presence of salt and glucose, has promising applications in several industrial processes due to its unique properties. Primarily, the enzyme's thermostability and alkaline tolerance suggest its potential use in the food and bioenergy industries^72^. The CelGH3_f17 β-glucosidases halotolerance and glucose enhancement up to a certain threshold is an advantageous trait not commonly observed in β-glucosidases^58^. Its tolerance to high glucose concentrations is particularly valuable in biofuel production, as it overcomes the common challenge of product inhibition in this process^63,64^. In the food industry, glucose tolerance β-glucosidase can play a vital role in processing, particularly in the degradation of cellulose or soy isoflavone in organic solvents^73^.

## Conclusion

In this study we cloned, expressed, and characterised a β-glucosidase, CelGH3_f17, from a previously constructed metagenomics libraries derived from an Ethiopian soda lake. This enzyme belongs to glycoside hydrolase family 3 (GH3) and performed optimum activity at 50 °C and pH 8.5. It was particularly resistant to high salt concentrations and glucose. Furthermore, the majority of metal ions and solvents examined showed little effect on its activity. Overall, CelGH3_f17 has favourable properties such as stability at alkaline pH, and high salt and glucose tolerance, making it a good choice for industrial applications, particularly in biomass degradation.

## Methods

### Screening and identification of CelGH3_f17 from a soda lake library

The CelGH3_f17 gene was identified from our previously constructed metagenomic libraries derived from Ethiopian soda lakes^37^. Briefly, metagenomics libraries were constructed from the water and sediment samples of the soda lakes. Following, functional screening of these libraries for β-glucosidase activity, clones exhibiting β-glucosidase activity were sequenced, and a comprehensive bioinformatics analysis were conducted. A sequence named CelGH3_f17 due to its success in cloning and high hit rate, was choosen for further investigation in this study.

### Sequence and structure analysis of CelGH3_f17 protein

Domain architecture and conserved domains of the CelGH3_f17 protein were identified with the Simple Modular Architecture Research Tool (SMART) program (http://smart.embl-heidelberg.de)^74^. To identify potential homologs of the CelGH3_f17 protein, we conducted a BLASTP search against the Protein Data Bank (PDB) using the National Center for Biotechnology Information (NCBI) platform, employing the default algorithm settings. The amino acid sequence of the CelGH3_f17 protein served as the query. Following this, the resulting FASTA sequences were incorporated into MEGA software^75^. The Neighbor-Joining method was employed to deduce the evolutionary history of these sequences. The three-dimensional (3D) structural model of CelGH3_f17 protein was predicted utilizing the Protein Homology/analogY Recognition Engine (Phyre2; V 2.0) web server^76^. This online program enabled the prediction of the tertiary structure of the CelGH3_f17 protein. Furthermore, ExPASy's Compute pI/MW tool^77^, was used to calculate the theoretical pI (isoelectric point) and MW (molecular weight) of the CelGH3_f17 protein.

### Cloning of the β-glucosidase gene

The CelGH3_f17 was synthesized and codon-optimized for expression in *E. coli* using Twist Bioscience's gene synthesis service. Briefly, the pET-28a (+) vector was prepared for cloning by digestion with BamHI and XhoI restriction enzymes, followed by purification. The synthesized CelGH3_f17 was similarly digested and purified. The gene was then ligated into the prepared vector using T4 DNA ligase, and the ligation mixture was transformed into chemically competent *E. coli* DH5α cells. Chemically competent DH5α cells were thawed gently on ice, and the ligation mixture with the CelGH3_f17 insert was added, followed by careful mixing to avoid cellular damage. The cells were then incubated on ice for 30 min to allow DNA adsorption, succeeded by a 45 s heat shock at 42 °C to increase membrane permeability and facilitate DNA uptake, and subsequently cooled on ice for 2 min. SOC medium was added to the transformation mixture for cell recovery, and the mixture was incubated at 37 °C with shaking for 1 h to enable antibiotic resistance gene expression. The transformed cells were then plated on LB agar plates containing kanamycin and incubated overnight at 37 °C. To confirm the β-glucosidase activity, the transformed cells were plated onto LB agar plates containing esculin hydrate (0.1%), ferric ammonium citrate (0.25%), and chloramphenicol (12.5 μg/ml). Colonies with β-glucosidase activity was identified by observing a surrounding black halo after 20-24 h incubation at 37 °C^78^.

### Expression and purification of the recombinant CelGH3_f17 protein

A single colony of transformant was inoculated into LB media supplemented with kanamycin (50 µg/ml) and incubated overnight at 37 °C with 220 rpm shaking. Then, this starter culture was used to inoculate a larger culture (1 L) at 1:50 dilution and again incubated at 37 °C with 220 rpm shaking. The expression was induced by adding 0.5 mM IPTG (Sigma); when the cell density reached an A_600_ of 0.4–0.6. The culture was then incubated for an additional 3.5 h at 37 °C. Ni–NTA resin (Qiagen) was used to purify the protein following the pET System manufacturer’s instructions. Briefly, an appropriate amount of Ni–NTA resin slurry was added to a gravity-flow column. Then, a binding buffer was passed through the column to equilibrate the resin. Thereafter, the filtered soluble protein was added to the equilibrated resin and incubated for 2 h at 4 °C. Then, after incubation, the flow-through was allowed to pass and the resin was washed with wash buffer three times. Finally, the purified protein bonded to the resin was eluted with an elution buffer*.* The purity and integrity of the protein were assessed by SDS-PAGE. Briefly, for SDS_PAGE analysis, each sample including IPTG-uninduced crude extract, IPTG-induced crude extract, and Ni–NTA chromatography purified extract, were mixed with SDS-PAGE loading buffer, heated at 95 °C for 5 min for denaturation, and then loaded onto an 12% SDS-PAGE gel. Electrophoresis was performed at a 120 voltage until the dye front reached the bottom of the gel. After electrophoresis, the gel was stained with Coomassie Brilliant Blue and destained to visualize protein bands.

### Enzyme activity assays

The β-glucosidase activity was measured by determining the hydrolysis of p-nitrophenyl-β-D-glucopyranoside (*p*NPG; Sigma) using the initial rate of accumulation of coloured reaction product as described by^9^. One hundred eighty microliter of 5 mM *p*NPG substrate was diluted in 50 mM Tris HCl buffer (pH 8.5) and mixed with a 20 μl aliquot of the acquired enzyme. Then, the mixture was incubated at 50 °C for 10 min, and the reaction was terminated by adding 100 μl of ice-cold 0.5 M Na_2_CO_3_. The release of p-nitrophenol (pNP) via enzymatic hydrolysis was indicated by the appearance of a yellow colour and the absorbance was measured with a UV/Vis microplate spectrophotometer (Multiskan GO, Thermo Scientific) at 405 nm. One unit (U) of β-glucosidase activity was defined as the amount of enzyme that released 1 μmol of pNP per minute from the substrate. All experiments were performed in three technical replicates. Statistical significance between treatments and a control group was assessed using post-hoc Tukey's Honestly Significant Difference (HSD) test. Differences were considered statistically significant at a p-value of < 0.05.

Relative activity is as follows:

$$\mathrm{Relative\,Activity }(\mathrm{\%}) = (\mathrm{Activity\,of\,sample }({\text{U}}) /\mathrm{ Maximum\,enzyme\,activity }({\text{U}})) * 100$$

### Characterization of the CelGH3_f17 protein

The effect of temperature on β-glucosidase activity was studied by assaying the enzyme in 50 mM Tris–HCl buffer (pH 8.5) at temperatures ranging from 30 to 80 °C. The effect of pH on β-glucosidase activity was investigated by incubating 180 µl of a substrate prepared at different pH buffers with 20 µl of an enzyme for 10 min at 50 °C. The substrate (5 mM *p*NPG) was prepared in 50 mM pH buffers ranging from 4.5 to 10.5. The buffers used were sodium acetate (pH 4.5–5.5), phosphate (pH 6.5), Tris–HCl (pH 7.5), and glycine–NaOH (pH 8.5–10.5). The stability of β-glucosidase at different temperatures was also evaluated. The enzyme was pre-incubated at temperatures of 37 °C, 50 °C, 60 °C, and 70 °C over a period of 16 h. Similarly, the pH stability of β-glucosidase was investigated by pre-incubating the enzyme in buffers with pH values of 7.5, 8.5, 9.5, and 10.5 at the optimal temperature for varying time points up to 16 h. After incubation, the residual activity of the enzyme was assessed as described above.

The effects of metal ions on the β-glucosidase activity were studied by introducing the metal ions (CuCl_2_, FeCl_3_, KCl, CaCl_2_, ZnCl_2_, MnCl_2_, MgSO_4_ and AlCl_3,_) into the reaction at a final concentration of 5 mM.

The effect of glucose on the activity of β-glucosidase was studied ranging from 0 to 500 mM concentrations. In the meantime, the effect of salt on the β-glucosidase activity was studied by supplementing salt, ranging from 0 to 4 M concentrations.

The effect of surfactants and inhibitors on β-glucosidase activity was studied by directly incorporating these in the reaction to a final concentration of 10 mM. The studied surfactants and inhibitors include mercaptoethanol, Ethylenediaminetetraacetic Acid (EDTA), Dimethyl SulfOxide (DMSO), Glycerol, Sodium Dodecyl Sulphate (SDS), and Triton X-100.

The substrates *p*NPG, CMC, and Xylan were used to study the specificity of substrate on β-glucosidase activity. The enzyme assay of the tested polysaccharides, CMC, and Xylan was based on the determination of reducing sugars by the dinitrosalicylic acid reagent (DNS) method^79^. One unit of cellulase activity was defined as the amount of enzyme capable of breaking down CMC to produce 1 µmol of glucose within a 1-min reaction. Similarly, one unit of xylanase activity was determined as the amount of enzyme that can release 1 µmol of xylose per minute reaction.

### Supplementary Information

Supplementary Table 1.

Supplementary Table 2.

## Data Availability

Sequence data that supports the findings of this study has been deposited in NCBI’s GenBank database and is publically available with an accession number of OR883674 (https://www.ncbi.nlm.nih.gov/nuccore/OR883674).
